# Breast cancer follow-up strategies in randomized phase III adjuvant clinical trials: a systematic review

**DOI:** 10.1186/1756-9966-32-89

**Published:** 2013-11-11

**Authors:** Isabella Sperduti, Patrizia Vici, Nicola Tinari, Teresa Gamucci, Michele De Tursi, Giada Cortese, Antonino Grassadonia, Stefano Iacobelli, Clara Natoli

**Affiliations:** 1 Unit of Biostatistics, Regina Elena National Cancer Institute, Rome 00144, Italy; 2 Division of Medical Oncology B, Regina Elena National Cancer Institute, Rome 00144, Italy; 3 Medical Oncology Unit, Department of Experimental and Clinical Sciences, University “G. d’Annunzio”, Chieti 66013, Italy; 4 Department of Oncology, “S.S. Trinita`” Hospital, Sora (FR) 00039, Italy; 5 Department of Oncology, “S.S. Annunziata” Hospital, Chieti 66013, Italy

**Keywords:** Breast cancer, Follow-up, Phase III clinical trial, Systematic review

## Abstract

The effectiveness of different breast cancer follow-up procedures to decrease breast cancer mortality are still an object of debate, even if intensive follow-up by imaging modalities is not recommended by international guidelines since 1997. We conducted a systematic review of surveillance procedures utilized, in the last ten years, in phase III randomized trials (RCTs) of adjuvant treatments in early stage breast cancer with disease free survival as primary endpoint of the study, in order to verify if a similar variance exists in the scientific world. Follow-up modalities were reported in 66 RCTs, and among them, minimal and intensive approaches were equally represented, each being followed by 33 (50%) trials. The minimal surveillance regimen is preferred by international and North American RCTs (P = 0.001) and by trials involving more than one country (P = 0.004), with no relationship with the number of participating centers (P = 0.173), with pharmaceutical industry sponsorship (P = 0.80) and with trials enrolling > 1000 patients (P = 0.14). At multivariate regression analysis, only geographic location of the trial was predictive for a distinct follow-up methodology (P = 0.008): Western European (P = 0.004) and East Asian studies (P = 0.010) use intensive follow-up procedures with a significantly higher frequency than international RCTs, while no differences have been detected between North American and international RCTs. Stratifying the studies according to the date of beginning of patients enrollment, before or after 1998, in more recent RCTs the minimal approach is more frequently followed by international and North American RCTs (P = 0.01), by trials involving more than one country (P = 0.01) and with more than 50 participating centers (P = 0.02). It would be highly desirable that in the near future breast cancer follow-up procedures will be homogeneous in RCTs and everyday clinical settings.

## Introduction

In the last years, a substantial increase in the number of women surviving breast cancer [1], the most frequent female cancer in the world [2-5], has been reported. This leads to the necessity to focus on breast cancer follow-up procedures for the high relevance they have for both patients and professional personnel [6]. The primary aim of routine post-operative surveillance after early stage breast cancer surgery, referred to as 'follow-up’, is to enhance survival, psychosocial and physical well-being of patients. The effectiveness of different breast cancer follow-up procedures for early detection of metastatic disease is an old issue, starting in the 1980s [7-10]. In the 1990s, evidences from phase III randomized trials (RCTs) demonstrated that intensive follow-up procedures do not improve outcome or quality of life when compared to patients’ educations about symptoms referral and regular physical examinations [11-18]. Nowadays, there is a general agreement on the utility of yearly mammography for detecting local recurrences and/or second primary cancers while intensive follow-up practices by imaging techniques (i.e. chest radiograph, bone scan and liver sonography) are not recommended by current international guidelines [19,20]. Nevertheless**,** the appropriateness of screening tests to be used as well as the frequency of follow-up procedures and the optimal follow-up duration are still object of debate [21-24], which reflects in the wide use of intensive surveillance and in the long-term follow-up period in everyday clinical practice [6,25-28].

Based on these premises, we conducted a systematic review of the surveillance procedures utilized in phase III RCTs of adjuvant treatments in early stage breast cancer in order to asses if a similar variance exists in the scientific world.

## Methods

### Literature search and eligibility criteria

We searched PubMed (PubMed, available at URL: http://www.ncbi.nlm.nih.gov/pubmed) from January 1, 2002 to December 31, 2012 for phase III RCTs of early breast cancer medical adjuvant therapies with disease free survival (DFS) as primary endpoint of the study [29]. We selected only full text publications (not abstracts), written in English-language. Trials on neoadjuvant therapies, neoadjuvant followed by adjuvant therapies, adjuvant bisphosphonates alone, non medical treatments, radiation therapies, adjuvant chemotherapy for loco-regional relapses and non-phase III trials were excluded. When multiple publications of the same RCT were identified, the first publication was selected. We used as keywords: *breast cancer adjuvant therapy, clinical trial, phase III, phase 3 and randomized.*

### Data extraction

Information extracted from each trial included: date of beginning of patients enrollment, geographic location, number of participating countries, sponsorship by pharmaceutical companies, number of participating centers, number of enrolled patients, follow-up description (modalities, frequency and duration). Follow-up was classified as minimal when only history/physical examination and/or automated blood chemistry studies, and intensive when chest radiographs ± bone scan ± liver sonography ± tumor markers were included. Screening and data extraction were performed independently by two investigators.

### Statistics

Descriptive statistics were used to report relevant study information. The associations between variables and follow-up data were tested by the Pearson’s chi-square test or Fisher’s exact test, as appropriate. All *p* values are reported as 2-sided and *p* values less than 0.05 denotes statistically significant association. A multiple correspondence analysis (MCA), an exploratory multivariate statistical technique, was used to analyze possible relationships among all variables and identify specific profiles [30]. In the MCA, associations between variables are displayed graphically as maps, and their position in the graphic is exclusively informative. The prediction of follow-up procedures was evaluated using a stepwise multivariate logistic regression. The cut-off p value for inclusion or exclusion in the model was set at 0.10 and 0.15, respectively. The Odds Ratio (OR) and the 95% confidence intervals (95% CI) were estimated for each variable. The SPSS software (SPSS version 19.0, SPSS Inc., Chicago, Illinois, USA) was used for all statistical evaluations.

## Results

**Figure 1:**
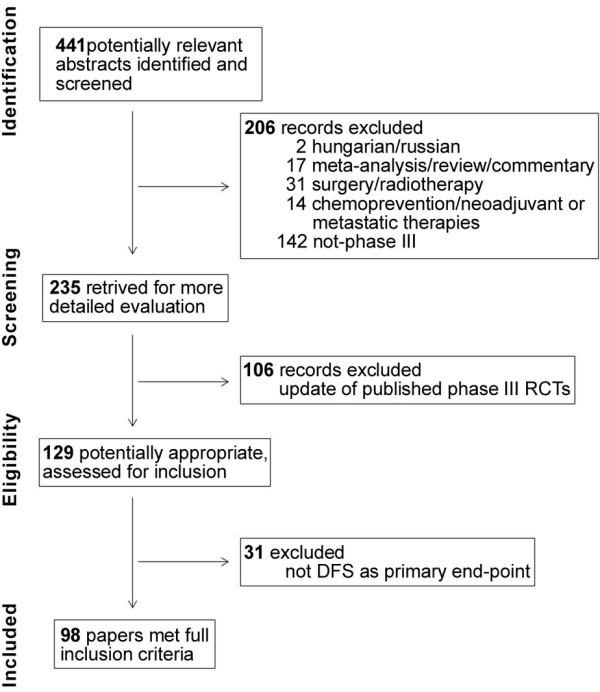
Flowchart of study selection.

Of 441 potentially relevant abstracts identified, 98 papers met full inclusion criteria: follow-up modalities were reported in 66 RCTs [31-95] while no information was given in the remaining 32 [96-127]. Two different trials, the ABCSG trial 8 and ARNO 95 trial, are reported in the same paper by Jakesz et al. [58]. The flowchart of search strategy is shown in Figure 1.

As shown in Table 1, there is a trend towards more frequently describing surveillance procedures in papers from international, West European or East Asian (Japan, Vietnam and China) RCTs than in those from North American (USA and Canada) RCTs (P = 0.06); no relationship has been found between other variables taken into account and the availability of follow-up data.

**Table 1:** Description of follow-up procedures in RCTs

	**Follow-up data**		**P value**
	**Yes**	**NO**	
	**No. (%)**	**No. (%)**	
**Geographic location**			
International	13 (68)	6 (32)	**0.06**
North America (USA and Canada)	10 (48)	11 (52)	
Western Europe	38 (79)	10 (21)	
East Asia (Japan, Vietnam, China)	5 (56)	4 (44)	
**Number of participating countries**			
1 country+	43 (66)	22 (34)	**0.49**
> 1 country	23 (74)	8 (26)	
**Number of participating centers**			
≤ 50	29 (81)	7 (19)	**0.75**
> 50	17 (77)	5 (23)	
**Industry sponsorship**			
Yes	37 (75)	12 (25)	**0.64**
No	29 (69)	13 (31)	
**Number of enrolled patients**			
≤ 1000 patients	34 (76)	11 (24)	**0.14**
> 1000 patients	32 (62)	20 (38)	

Legends: RCTs = randomized clinical trials.

Among the 66 papers describing follow-up methodology, minimal and intensive approaches were equally represented, each being followed by 33 (50%) trials. Only 6 papers report the use of tumor markers measurement (carcinoembryonic antigen and carbohydrate antigen 15–3) during follow-up [46,48,57,75,82,88] and none includes the use of computed tomography scans, positron emission tomography scanning and magnetic resonance imaging.

Table 2 shows that the minimal surveillance regimen is preferred by international and North American RCTs (P = 0.001) and by trials involving more than one country (P = 0.004), while there is no relationship with the number of participating centers (P = 0.173), the pharmaceutical industry sponsorship (P = 0.80), trials enrolling > 1000 patients (P = 0.14). Breast cancer follow-up guidelines, recommending the minimal approach, were published by the American Society of Clinical Oncology in 1997 [128]. Interestingly, no differences in follow-up modalities have been detected in RCTs enrolling patients before and after 1998 (P = 0.58). Stratifying data according to the date of beginning of patients enrollment (i.e. before or after 1998), even if numbers are small, in more recent studies there is a higher use of the minimal approach by international and North American RCTs (P = 0.01) and by trials involving more than one country (P = 0.01), and more than 50 participating centers (P = 0.02), with a trend toward statistical significance for trials enrolling > 1000 patients (P = 0.06) (Table 3).

**Table 2:** Follow-up methodologies in RCTs

	**Follow-up Approach**		**P value**
	**Minimal**	**Intensive**	
	**No. (%)**	**No. (%)**	
**Geographic location**			
International	12 (92)	1(8)	**0.001**
North America (USA and Canada)	7 (70)	3 (30)	
Western Europe	13 (34)	25 (66)	
East Asia (Japan, Vietnam, China)	1 (20)	4 (80)	
**Number of participating countries**			
1 country	16 (37)	27 (63)	**0.004**
> 1 country	17 (74)	6 (26)	
**Number of participating centers**			
≤ 50	11 (38)	18 (62)	**0.173**
> 50	10 (59)	7 (42)	
**Industry sponsorship**			
Yes	18 (49)	19 (51)	**0.80**
No	15 (52)	14 (48)	
**Number of enrolled patients**			
≤ 1000 patients	14 (41)	20 (58)	**0.14**
> 1000 patients	19 (59)	13 (41)	
**Date of beginning of patients enrollment**			
From 1981 to 1997	23 (48)	25 (52)	**0.58**
From 1998 to 2002	10 (56)	8 (44)	

Legends: RCTs = randomized clinical trials.

**Table 3:** Follow-up methodologies in RCTs according to the date of beginning of patients enrollment

	**Date of beginning of patients enrollment**					
	**Before 1998**			**After 1998**		
	**Follow-up approach**			**Follow-up approach**		
	**Minimal**	**Intensive**		**Minimal**	**Intensive**	
	**No. (%)**	**No. (%)**	**P value**	**No. (%)**	**No. (%)**	**P value**
**Geographic location**						
International	7 (87)	1 (13)		5 (100)	**-**	**0.01**
North America (USA and Canada)	3 (60)	2 (40)		4 (80)	1 (20)	
Western Europe	12 (37)	20 (63)		1 (16)	5 (83)	
East Asia (Japan, Vietnam, China)	1 (33)	2 (67)	**0.07**	-	2 (100)	
**Number of participating countries**						
1 country	13 (39)	20 (60)		3 (30)	7 (70)	**0.01**
> 1 country	10 (66)	5 (33)	**0.08**	7 (87)	1 (87)	
**Number of participating centers**						
≤ 50	11 (46)	13 (54)		**-**	5 (100.0)	**0.02**
> 50	6 (54)	5 (46)	**0.63**	4 (67)	2 (33)	
**Industry sponsorship**						
Yes	9 (41)	13 (59)		9 (60)	6 (40)	**0.40**
No	14 (54)	12 (46)	**0.37**	1 (33)	2 (67)	
**Number of enrolled patients**						
≤ 1000 patients	13 (45)	16 (55)		1 (20.0)	4 (80.0)	**0.06**
> 1000 patients	10 (53)	9 (47)	**0.60**	9 (69)	4 (31)	

**Figure 2:**
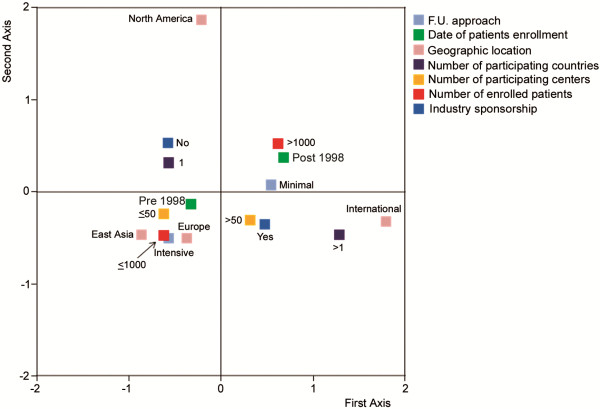
Multiple correspondence analysis of possible relationships among all variables.

The graphical map of MCA (Figure 2) shows that intensive follow-up procedures cluster with Western European and East Asian studies, studies with less than 50 participating centers and less than 1000 enrolled patients, and with patients enrollment beginning before 1998, while the minimal approach clusters with RCTs enrolling more than 1000 patients and beginning enrollment after 1998 (Figure 2). At multivariate regression analysis, only geographic location of the trial was predictive for a distinct follow-up methodology (P = 0.008). In particular, setting as a reference the international studies, Western European (P = 0.004) and East Asian studies (P = 0.010) use intensive follow-up procedures with a significantly higher frequency than international RCTs, while no differences are detected between North American and international RCTs.

For each follow-up approach, the frequency at which the different exams are performed is highly variable, ranging from 1 to 4 times/year for history and/or physical examinations, and from 1 to 3 times/year for imaging modalities, as shown in Table 4. Almost all RCTs showed the highest number of evaluations/year in the first 1–2 years of follow-up; 5-year follow-up and annually thereafter was chosen by almost all studies, with the following exceptions: two studies interrupted all imaging modalities at the 3rd year [83,84]; one study discontinued chest radiographs and bone scan at the 4th year [46] and one study ended chest radiographs at the 3rd year [66].

**Table 4:** Frequency of different exams from year 1 to 5 of follow-up

**Variable**		**1° year**		**2° year**		**3° year**		**4° year**		**5° year**	
		**Min_ Follow-up**	**Int_ Follow-up**	**Min_ Follow-up**	**Int_ Follow-up**	**Min_ Follow-up**	**Int_ Follow-up**	**Min_ Follow-up**	**Int_ Follow-up**	**Min_ Follow-up**	**Int_ Follow-up**
		**times/year**	**times/year**	**times/year**	**times/year**	**times/year**	**times/year**	**times/year**	**times/year**	**times/year**	**times/year**
History/physical examination 46 RCTs	Median	4.0	4.0	2.0	4.0	2.0	2.0	2.0	2.0	2.0	2.0
	Lower-Higher limit	1.0-4.0	1.0-4.0	2.0-4.o	1.0-4.0	1.0-2.0	1.0-4.0	2.0	1.0-4.0	1.0-2.0	1.0-4.0
Physical examination 18 RCTs	Median	3.0	3.5	2.5	3.0	2.0	2.5	2.0	2.0	2.0	2.0
	Lower-Higher limit	1.0-4.0	3.0-4.0	1.0-4.0	2.0-4.0	2.0-4.0	3.0-4.0	1.0-4.0	1.0-3.0	1.0-4.0	1.0-3.0
Chest radiograph 33 RCTs	Median		1.0		1.0		1.0		1.0		1.0
	Lower-Higher limit		1.0-3.0		1.0-3.0		1.0-3.0		1.0-2.0		1.0-2.0
Bone scan 19 RCTs	Median		1.0		1.0		1.0		1.0		1.0
	Lower-Higher limit		1.0-3.0		1.0-3.0		1.0-3.0		1.0-3.0		1.0-2.0
Liver sonography 24 RCTs	Median		1.0		1.0		1.0		1.0		1.0
	Lower-Higher limit		1.0-3.0		1.0-3.0		1.0-3.0		1.0-2.0		1.0-2.0

Legends: Min_ = minimal; Int_ = intensive.

## Discussion

The results of our systematic review demonstrates that among phase III RCTs of adjuvant therapies for early stage breast cancer, minimal and intensive follow-up approaches are equally used. However, it should be noted that not all the papers, mainly from North America, report the modalities of follow-up [91-121], even if we selected RCTs with primary endpoint represented by DFS, which can be affected by the surveillance methodologies applied. Possible explanations could be that *i)* the authors and referees do not think this is a relevant issue or *ii)* a follow-up according to established guidelines was applied, thus making it unnecessary to specify. The second hypothesis may be more likely, since the minimalist follow-up suggested by international guidelines is more frequently followed by North American while intensive follow-up is preferred by Western European and East Asian trialists.

Our analysis also suggests that the use of the different strategies of follow-up is not dictated by the necessity of costs containment as it has been suggested [129-131], since no relationship with industrial sponsorships, number of participating centers and number of enrolled patients has been found. It seems more likely that the intensive surveillance methodology in RCTs follows Western European and East Asian cultural attitudes of scientists and medical oncologists towards the care of breast cancer patients [132]. In this respect, it has recently been reported that many European and East Asian breast cancer patients receive more intensive follow-up care than recommended by the current guideline [6,25,26,133,134] even if, at a lesser extent, this has been also reported for American and Canadian patients [27,28].

The frequency of follow-up is higher in the first 2–3 years after surgery and tends to decrease thereafter. Almost all RCTs, except few studies [46,83,84], continue programmed controls at least 5 years after treatment, independently from the chosen follow-up methodology. These issues are still object of debate [135], since neither the optimum frequency nor duration of follow-up has been clearly defined [23,136,137].

Results from two Italian phase III RCTs, both published in 1994 [11,12] and several retrospective studies [138-141] demonstrated that intensive follow-up strategies including chest radiography, bone scan, liver ultrasound and tumor markers measurements do not improve survival as compared to history taking, physical examinations and annual mammography. On the basis of these data, the American Society of Clinical Oncology published in 1997 and periodically updated thereafter [19,128,142] breast cancer follow-up guidelines recommending a minimal approach. We found no increase in the use of minimalist follow-up among RCTs beginning to enroll patients one year after published guidelines (i.e. 1998). However, more recently the minimal approach is being preferred by most international and North American RCTs, and bigger trials, such as those involving more than one country and more than 50 participating centers. It is relevant to point up that the use of the intensive follow-up is still present in almost 45% of new generation RCTs.

A possible limit of our study may be represented by the choice of studies written in English, although the vast majority of RCTs are currently published in this language and in scientific journal indexed in PubMed. In addition, it should be underlined that it is likely the statistic analysis could be not completely reliable, considering that in some of the subcategories considered in the study, the number of eligible RCTs is low.

## Conclusions

Current breast cancer follow-up guidelines, which are based on RCTs, suggest a minimal follow-up approach for surveillance of early breast cancer patients, but this suggestion is not widely applied neither in phase III RCTs of adjuvant treatments nor in real world clinical practice. Whether the minimal follow-up approach will still be the recommended option in the future, is to be confirmed. In fact, more effective and sophisticated diagnostic procedures may be useful to point out severe long-term side effects of new molecularly targeted agents as well as an early detection of oligometastatic disease might be suitable for cure with newer therapeutic strategies, as it has been suggested for other neoplasms [143]. Finally, it would be highly desirable that in the near future the follow-up procedures will be homogeneous in RCTs and everyday clinical settings.

## Abbreviations

DFS: Disease free survival; MCA: Multiple correspondence analysis; OR: Odds ratio; RCTs: Randomized clinical trials.

## Competing interests

The authors have no potential conflicts of interest to declare.

## Authors’ contributions

IS supervised the data collection, performed the statistical analyses and revised the manuscript; AG, MDT and GC performed literature search and data extraction; NT and TG wrote the manuscript; PV and SI critically revised the manuscript; CN conceived the study and critically revised the manuscript. All authors read and approved the final manuscript.
